# Temporal Integration of Text Transcripts and Acoustic Features for Alzheimer's Diagnosis Based on Spontaneous Speech

**DOI:** 10.3389/fnagi.2021.642647

**Published:** 2021-06-14

**Authors:** Matej Martinc, Fasih Haider, Senja Pollak, Saturnino Luz

**Affiliations:** ^1^Department of Knowledge Technologies, Jozef Stefan Institute, Ljubljana, Slovenia; ^2^Usher Institute, Edinburgh Medical School, The University of Edinburgh, Edinburgh, United Kingdom

**Keywords:** Alzheimer's dementia detection, speech, language, acoustic features, lexical features, natural language processing, speech processing, machine learning

## Abstract

**Background:** Advances in machine learning (ML) technology have opened new avenues for detection and monitoring of cognitive decline. In this study, a multimodal approach to Alzheimer's dementia detection based on the patient's spontaneous speech is presented. This approach was tested on a standard, publicly available Alzheimer's speech dataset for comparability. The data comprise voice samples from 156 participants (1:1 ratio of Alzheimer's to control), matched by age and gender.

**Materials and Methods:** A recently developed Active Data Representation (ADR) technique for voice processing was employed as a framework for fusion of acoustic and textual features at sentence and word level. Temporal aspects of textual features were investigated in conjunction with acoustic features in order to shed light on the temporal interplay between paralinguistic (acoustic) and linguistic (textual) aspects of Alzheimer's speech. Combinations between several configurations of ADR features and more traditional bag-of-n-grams approaches were used in an ensemble of classifiers built and evaluated on a standardised dataset containing recorded speech of scene descriptions and textual transcripts.

**Results:** Employing only semantic bag-of-n-grams features, an accuracy of 89.58% was achieved in distinguishing between Alzheimer's patients and healthy controls. Adding temporal and structural information by combining bag-of-n-grams features with ADR audio/textual features, the accuracy could be improved to 91.67% on the test set. An accuracy of 93.75% was achieved through late fusion of the three best feature configurations, which corresponds to a 4.7% improvement over the best result reported in the literature for this dataset.

**Conclusion:** The proposed combination of ADR audio and textual features is capable of successfully modelling temporal aspects of the data. The machine learning approach toward dementia detection achieves best performance when ADR features are combined with strong semantic bag-of-n-grams features. This combination leads to state-of-the-art performance on the AD classification task.

## 1. Introduction

While the natural history of Alzheimer's Disease (AD) and the form of dementia it causes are mainly characterised by memory impairment, a wide range of cognitive functions are known to be affected by the process of neurodegeneration triggered by the disease. Several standardised neuropsychological tests are currently employed to detect such impairments for the purposes of diagnosis and assessment of disease progression. However, these tests often take place in clinics and consist of constrained cognitive tasks, where the patient's performance may be affected by extraneous factors such as variations in mood, poor sleep the night before the test, etc. Recent progress in artificial intelligence (AI) and machine learning (ML) technology has opened new avenues for more comprehensive monitoring of cognitive function, and tests based on spontaneous speech and language data have emerged as possible tools for diagnostic and prognostic assessment (de la Fuente Garcia et al., 2020; Petti et al., 2020). In this paper we investigate the hypothesis that integration of acoustic and textual data into a unified ML model enhances the accuracy of AD detection. Specifically, we present a model that integrates acoustic and textual modalities on a temporal (i.e., time-based) dimension. The motivation for doing so arises from the nature of the task used to elicit the speech data used in this study. These data consist of spontaneous speech elicited through the Cookie Theft description task from the Boston Diagnostic Aphasia Exam (Goodglass et al., 2001), which involves visuospatial as well as verbal ability.

Along with language, visuospatial function is affected early in AD. This is manifested in the form of non-salience of visual input stimulus, and degraded attentional focus and visual search, among other disturbances (Cronin-Golomb, 2011). Using a similar picture description task, Meguro et al. (2001) observed hemispatial visual searching impairment in some participants with AD, in correlation with decreased contralateral parietal blood flow. Other studies involving picture descriptions have associated AD with simultanagnosia, a disorder of attentive exploration of the spatial field (Vighetto, 2013). They found that persons with AD tended to produce “slow and partial [descriptions], one detail after the other, without ability to capture a global perception of the drawing.” Our assumption is that such disturbances of visuospatial function will be reflected in differences in temporal order between the descriptions produced by participants with AD and those produced by non-AD participants. As Cummings (2019) observed, while the Cookie Theft picture is a static scene, causal and temporal relations can be inferred from the various elements depicted in it. Capturing these relations is necessary to give a complete description of the picture, as “certain events in the scene must take place before other events in order for a description of the picture to make sense.” If, as seems likely, degraded attention focus hinders the participant's ability to identify such events, one should expect the temporal organisation of events in the scene description to differ in AD.

We therefore propose an approach to speech and language which incorporates temporal information. Unlike most other approaches, where content is represented as order-agnostic features with at most short distance dependencies, our model accounts for temporal aspects of both linguistic and acoustic features. We employ our recently developed Active Data Representation (ADR) processing technique (Haider et al., 2020) and present a novel way of fusing acoustic and text features at sentence and word level. We show that these features are capable of modelling temporal aspects of text and audio, but fall short of semantic modelling. To address this shortcoming, we propose combining ADR features with term frequency-inverse document frequency weighted bag-of-n-grams features, which proved effective in modelling semantics in previous studies (Martinc and Pollak, 2020). The final combination of ADR and bag-of-n-grams features leads to state-of-the-art performance on the AD classification task^1^.

## 2. Related work

The complex multimodal ways in which AD symptoms may appear calls for increasingly interdisciplinary research (Turner et al., 2020). Current research on AD involves not only biomedicine, neuroscience, and cognitive psychology, but also increasingly AI and machine learning methods. Studies connecting language and AD have focused mostly on formal aspects of language (i.e., lexicon, syntax and semantics), but the analysis of continuous speech has been progressively seen by researchers as a source of information that may support diagnosis of dementia and related conditions (Lopez-de Ipiña et al., 2015, 2016; Luz et al., 2018; Toth et al., 2018; Haulcy and Glass, 2021; Mahajan and Baths, 2021).

Language research into AD has employed high-level features such as information content, comprehension of complexity, picture naming and word-list generation as predictors of disease progression (Reilly et al., 2010). A study by Roark et al. (2011) used natural language processing (NLP) and automatic speech recognition (ASR) to automatically annotate and time-align a few spoken language features (pause frequency and duration), and compared these methods to manual analysis. They analysed audio recordings of 74 neuropsychological assessments to classify mild cognitive impairment (MCI) and healthy elderly participants. Their best classifier obtained an area under the receiver operating curve (AUC) of 86% by including a combination of automated speech and language features and cognitive tests scores. Jarrold et al. (2014) worked with a dataset consisting of semi-structured interviews from 9 healthy participants, 9 with AD, 9 with frontotemporal dementia, 13 with semantic dementia, and 8 with progressive nonfluent aphasia. With an automatic speech recognition (ASR) system, they extracted 41 features, including speech rate, and the mean and standard deviation of the duration of pauses, vowels, and consonants. They used a multilayered perceptron network, achieving an accuracy of 88% for AD vs. healthy subjects based on lexical and acoustic features. A more recent study by Luz et al. (2018) extracted graph-based features encoding turn-taking patterns and speech rate (Luz, 2009) from the Carolina Conversations Collection (Pope and Davis, 2011) of spontaneous interviews of AD patients and healthy controls. Their additive logistic regression model obtained 85% accuracy in distinguishing dialogues involving an AD speaker from controls.

More recently, multimodal representations have been explored, combining linguistic and paralinguistic aspects of communication (Haider et al., 2020; Mahajan and Baths, 2021), as well as eye-tracking and other sensor modalities (Jonell et al., 2021). Those studies combined signal processing and machine learning to detect subtle acoustic signs of neurodegeneration which may be imperceptible to human diagnosticians. Toth et al. (2018), for instance, found that filled pauses (sounds like “hmmm,” etc.) could not be reliably detected by human annotators, and that detection improved by using ASR-generated transcriptions. Using ASR features with a random forest classifier, Toth et al. (2018) reported an improvement over manually generated features (75 vs. 69.1% accuracy) for AD detection. Similar machine learning methods were used by König et al. (2015), who reported an accuracy of 79% when distinguishing MCI participants from healthy controls; 94% for AD vs. healthy; and 80% for MCI vs. AD. However, their tests involved different data collection procedures, including semantic fluency and sentence repetition tasks, in addition to a picture description task, with most features extracted from non-spontaneous, non-connected speech data. Motivated by the prospect of comprehensive cognitive status monitoring (Luz, 2017), studies in this field have moved toward analysis of spontaneous speech, and toward languages other than English. Weiner et al. (2016) analysed semi-structured German dialogues employing linear discriminant analysis to classify participants as healthy controls, Alzheimer's or age-associated cognitive decline, obtaining a mean accuracy score of 85.7%. This work has later been extended for prediction of development of dementia within 5 and 12 years in participants of the Interdisciplinary Longitudinal Study on Adult Development And Aging (ILSE), using a combination of acoustic and linguistic features (Weiner et al., 2019). Others have investigated the use of virtual agents as a data collection strategy for AD detection. Tanaka et al. (2017) collected dialogue, eye-tracking and video data from 29 Japanese participants who conducted structured dialogues with a virtual agent. They obtained 83% accuracy in classifying AD and control participants, using combined acoustic and textual modalities on a support vector machine (SVM) classifier. Mirheidari et al. (2019a) compared the accuracy of automated conversational analysis (ML with a combination of acoustic and linguistic features) for detection of AD on recorded doctor-patient consultations and on dialogues recorded through human-robot interaction. They reported similar accuracy for both settings using manual transcriptions (≈90%), suggesting that automated dialogue collection could be useful in mental health monitoring.

These studies evidence the heterogeneity with which language and speech impairments are displayed in AD and related diseases. Duong et al. (2005) ran a cluster analysis with data from picture narratives and concluded that, rather than a common profile, there were several discourse patterns that could be indicative of differences between healthy ageing and AD. This heterogeneity seems to be more evident in AD than in specific disorders such as primary progressive aphasia (Ahmed et al., 2013), especially in early stages of AD (Hodges and Patterson, 1995). Therefore, we hypothesise that a comprehensive analysis of state-of-the-art paralinguistic feature sets which have been successfully used in different prediction tasks may help identify such patterns and enhance accuracy of early AD detection.

The Pitt Corpus (Becker et al., 1994), which forms part of the DementiaBank (MacWhinney, 2019), and more specifically its Cookie Theft test sub-corpus, remains one of the very few available datasets to link spontaneous speech from dementia patients and healthy controls (recordings and transcriptions) with clinical information. Therefore, this dataset has been used in several studies, including the studies by Fraser et al. (2016); Hernández-Domínguez et al. (2018), and others (Yancheva and Rudzicz, 2016; Luz, 2017; Orimaye et al., 2017; Guo et al., 2019; Mirheidari et al., 2019b; Haider et al., 2020). These studies used different combinations of information coverage measures, linguistic features and acoustic features for automatic classification of dementia under different representation methods, ranging from simple descriptive statistics to more complex feature embedding representations. Among these studies, only Mirheidari et al. (2019b) investigated the possible relation, which we discussed above, between the temporal organisation of picture descriptions and cognitive impairment. In that work, verbal references were used to simulate the participants gaze and extract features corresponding to “areas of interest.” By combining such features with timing and pause information, and GloVe word vectors (Pennington et al., 2014) they were able to achieve 80% *F*_1_ score on manually transcribed data, and *F*_1_ = 72% on ASR outputs.

Speech research aiming at dementia detection is heterogeneous and comparisons are difficult to draw. Heterogeneity of dataset hinders comparison among the various studies on spontaneous speech for AD detection. The ADReSS challenge dataset (Luz et al., 2020) was created to mitigate this problem. In the shared task posed by ADReSS, all participants used the same dataset, which was balanced for age and gender and acoustically normalised. This is the dataset used in the present study. The various approaches proposed to tackle the ADReSS challenge included state-of-the-art deep learning and word embedding methods, and focused mainly on linguistic features extracted from the manually generated transcripts. The winning team (Yuan et al., 2020) leveraged audio recordings to obtain information about pauses in speech, encoding them as punctuation. The modified transcripts with encoded pauses were fed into an ensemble of 50 BERT (Devlin et al., 2019) and 50 ERNIE (Zhang et al., 2019) models, and majority voting was employed to derive the final predictions on the test set. They reported best accuracy (89.58%) when an ensemble of 50 ERNIE models was applied.

## 3. Dataset

This study uses the ADReSS subset of the Pitt Corpus, derived from a dataset gathered longitudinally between 1983 and 1988 on a yearly basis as part of the Alzheimer Research Program at the University of Pittsburgh (Becker et al., 1994; Corey Bloom and Fleisher, 2000), and made available through DementiaBank (MacWhinney, 2019). Participants are categorised into three groups: dementia, control (non-AD), and unknown status. All participants were required to be above 44 years of age, have at least 7 years of education, have no history of nervous system disorders or be taking neuroleptic medication, have an initial Mini-Mental State Examination (MMSE) score of 10 or more and be able to provide informed consent. Extensive neuropsychological and physical assessments conducted on the participants are also included (Becker et al., 1994).

While the Pitt Corpus contains data elicited through several tasks, our selected subset exclusively used the Cookie Theft description task subset, where participants were asked to describe the Cookie Theft picture from the Boston Diagnostic Aphasia Examination (Becker et al., 1994; Goodglass et al., 2001). This study specifically uses a subset of AD and control data matched for age and gender provided by the ADReSS challenge (Luz et al., 2020) to avoid bias, guarantee repeatability, and allow direct comparison with other ML approaches. In the following section, we provide a brief description of the methods used in the generation of the ADReSS dataset. The dataset and baseline results for the AD detection challenge are presented in detail by Luz et al. (2020).

### 3.1. The ADReSS Dataset

The pipeline employed in the preprocessing of the audio files is shown on the top part of Supplementary Figure 1. Initially, noise was sampled from short intervals from each audio recording, and subsequently spectral subtraction was applied to eliminate any noise matching those samples. Other non-target sounds such as background talk, ambulance sirens and door slamming, were minimised through selection of audio files with signal-to-noise ratio (SNR) ≥ −17 dB. Where multiple audio files existed per participant, the ADReSS organisers chose a subset that maximised audio quality and the number of samples in the matched dataset by selecting the latest recording, subjected to age and gender matching constraints. This resulted in a selection of 62 (≈ 40%) recordings taken on baseline visits, 57 (≈ 37%) on first visits, 19 (≈ 12%) on second visits, 17 (≈ 11%) on third visits and one (< 1%) on the fourth visit.

As age and gender are considered major risk factors for dementia (Dukart et al., 2011), these variables are possible confounders between the AD and non-AD groups. To eliminate this possible confounding, these groups are matched for age and gender in the ADReSS dataset. For age, 5-year ranges were chosen empirically to optimise the number of recordings included in the final dataset. As a result, 156 participants matched the inclusion criteria. Of these, 78 were healthy and 78 were diagnosed with probable AD. Supplementary Table 1 presents the demographics of the data used for training and testing. We note that the only patient in the [50, 55) age interval in the AD training set had an MMSE of 30, which would not normally match the diagnosis criterion for AD. Upon detailed inspection of the Pitt metadata we found that this patient in fact had an MCI diagnosis (memory only) and therefore should not have been included in the dataset. However, we decided to keep this data point in our training set for comparability with other models trained on the ADReSS dataset.

## 4. Temporal Analysis

**Figure 1:**
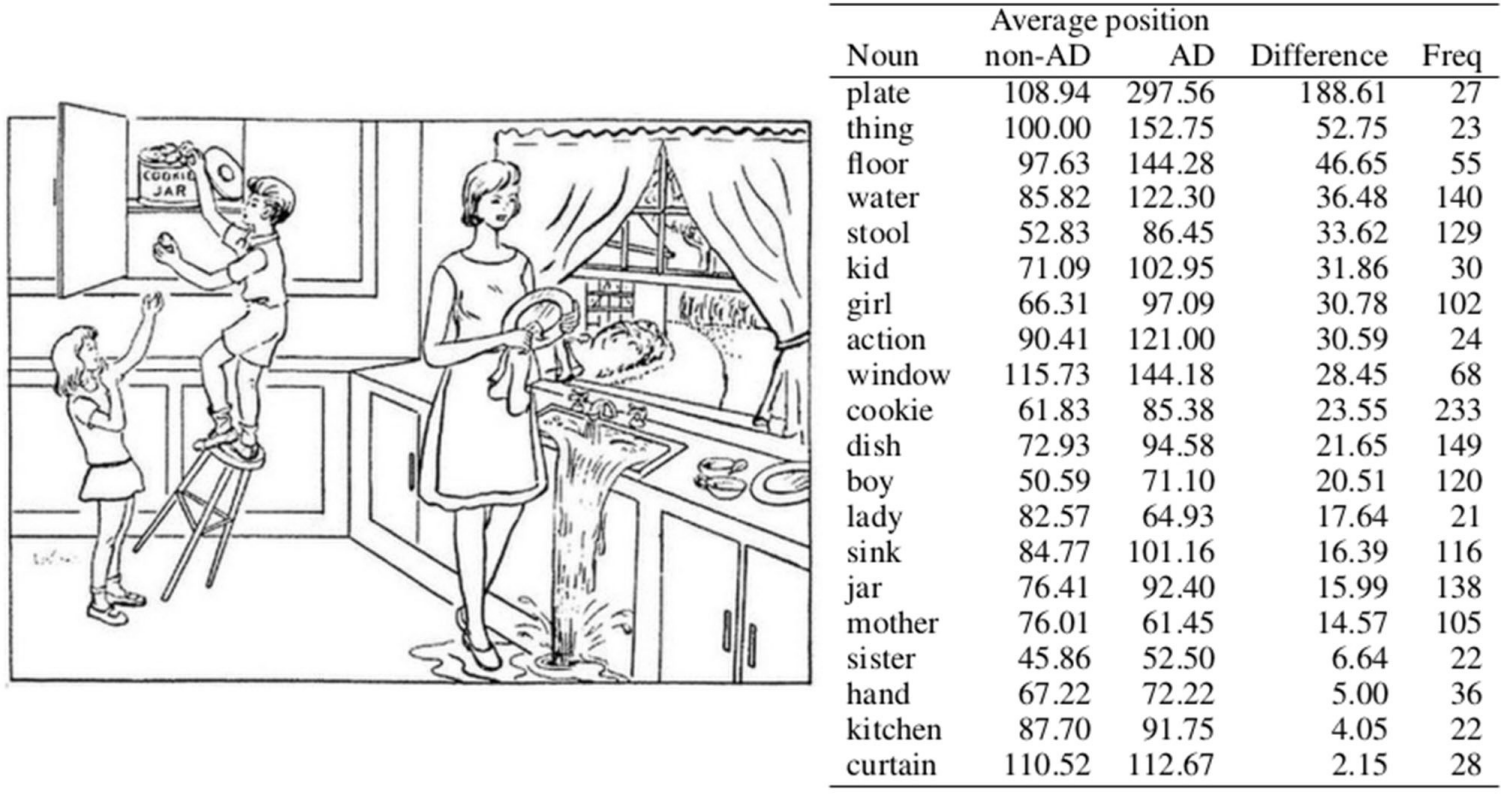
Average position of nouns that appear at least 20 times in the training set. AD and non-AD stand for average position in the speech transcripts of patients with AD and control group patients, respectively. Difference denotes the absolute difference between these averages, and Freq denotes the frequency of the noun in the corpus. The nouns are sorted according to the difference column.

As discussed in section 1, temporal aspects of the descriptions might provide important predictors in distinguishing between AD and non-AD speech. In this section, we present a temporal analysis of the transcripts, investigating the underlying assumption that the order in which specific situations in the cookie theft picture (see Figure 1) are described differs. More specifically, we investigate if there is enough information available for the models to detect the temporal discrepancies between the two diagnosis groups.

### 4.1. Training Set Analysis

In order to gain insight into whether the above hypothesis of temporal contrast between AD and non-AD patients is plausible, we conducted a statistical analysis on the training set, focusing on nouns, due to their function of denoting objects that can be easily connected to specific events in the image. Using the Stanza library (Qi et al., 2020) for assigning part-of-speech tags and lemmatisation, we extracted lemmas of nouns that appear at least 20 times in the test set. A threshold of 20 was used to filter out words used by a small minority of patients, which do not necessarily describe the events depicted in the picture. Since we are only interested in the differences between the target groups in regards to temporal aspect of the patient's description of the image, we also removed nouns that appear only in transcripts belonging to a certain group. This way we obtain a list of 20 nouns presented in Figure 1, which correspond to the constituents of the picture description task.

We determine a transcript position for each appearance of each noun (e.g., if the noun appears as the first word in the corpus, the position is one) and calculate an average noun position for each class, that is, the average of all positions of a specific noun in each class. The nouns in Figure 1 are sorted according to the difference between the average positions in each class.

One can see that the noun *plate*, for instance, has very different positions in descriptions produced by the distinct groups. It appears in sentences such as “*Two cups and a plate are on the counter there.”* and “*The lady is wiping a plate while the sink overflows”*., which are sentences describing details most likely not noticed by all participants (Cummings, 2019). Another noun with different positions is *thing*, which appears in sentences such as “*And the whole thing is going to collapse.”*, describing more than just one specific element or an action concerning several constituents in the picture. *Floor*, the noun with the third biggest difference between the average positions in each class on the other hand appears in sentences such as “*There's water on the floor.”* and “*And the stool is going to knock him on the floor.”*, and is related to more central parts of the action seen in the picture. While both AD patients and non-AD control group use these nouns to describe the picture and the actions related to these nouns, they appear to focus on them at different times in their descriptions.

The nouns at the end of the list are also interesting, since they denote situations in the picture described synchronously by both AD and non-AD patients. Noun *hand* is mostly related to a situation of the boy grabbing a cookie (e.g., “*He's grabbing a cookie in his hand.”*) or to a situation of the girl reaching for a cookie (e.g., “*And the girl's trying to help and she's reaching her hand up.”*). The noun *kitchen* appears in sentences such as “*Uh there's a set of kitchen cabinets.”*, mostly describing static elements in the image. Similarly can be said for the noun *curtain*, which mostly appears in sentences describing static elements (e.g., *Curtains at the window*.) but can nevertheless also appear in sentences describing some rather detailed observations (*e.g., “Curtains are blowing I think.”*).

### 4.2. Modelling Temporal Differences With Temporal Bag-of-Words

While the statistical analysis above offers some evidence of temporal differences in transcripts of AD and non-AD patients, a question remains as to whether classification models can detect these subtle differences. While assuming that they can is in our opinion a reasonable hypothesis, there is at least one reason to doubt this hypothesis. The presence of stronger features (i.e., semantic features, such as unigrams appearing only in one class) might cause the classifier to ascribe low importance to less subtle temporal aspects. Since in this section our focus is on ascertaining whether modelling of temporal aspects of the transcripts is possible rather than obtaining optimal accuracy (which is addressed in section 5.1), we can easily avoid this problem by restricting the classifier's model to contain only temporal features.

Therefore, we employed a simple bag-of-words model (Baeza-Yates and Ribeiro-Neto, 1999) to confirm or reject the hypothesis that the temporal differences between the non-AD and AD groups observed in the training set (see section 4.1) are relevant to the classification model. To track temporal order each transcript is divided into three sequential chunks of the same word length^2^. Words in each transcript belonging to the first chunk are given a suffix of _1, words belonging to the second chunk are given a suffix of _2, and words belonging to the third chunk are given a suffix of _3. This way the same words appearing in different sections of the transcript are distinguished by the bag-of-words model and we therefore obtain three features for each word, since in this bag-of-words model the same word with a different suffix is treated as a different word. Thus, we build a classifier that, rather than simply focusing on semantic differences (i.e., how many times a specific word appears in a specific transcript belonging to a specific group) also focuses on temporal differences (i.e., whether a specific word appears in a specific temporal chunk of a transcript belonging to a specific group). As we limited the word features in this model to the nouns appearing in Figure 1, the classifier is learnt to predict AD only on the basis of 60 features (i.e., 20 words from a list, each of them with three distinct suffixes according to the position in the text) derived from 20 nouns, which appear in transcripts of both AD and non-AD patients.

We used the same classification approach as in our classification experiments described in detail in section 5.1, that is, we trained and tested 50 random forest classifiers (Breiman, 2001) with 50 trees of maximum depth 5 by employing leave-one-out cross validation (LOOCV) on the training set, each time using a different random seed. The predictions of these models on the training set were then used for majority voting in order to derive final predictions^3^.

We measured the performance of the model by calculating accuracy according to the following equation:

(1) $$\begin{array}{r} accuracy=\frac{TP+TN}{TP+FP+TN+FN}, \end{array}$$

where TP stands for true positive examples (i.e., examples that the classifier correctly assigned to the AD class), TN stands for true negative examples (i.e., examples that the classifier correctly assigned to the non-AD class), FP stands for false positive examples (i.e., examples that the classifier incorrectly assigned to the AD class) and FN for false negative examples, which the classifier incorrectly assigned to the non-AD class.

The final majority voting accuracy for LOOCV is 77.78%, which indicates that the model is capable of successfully leveraging temporal differences. The Scikit-learn library (Pedregosa et al., 2011) implementation of the algorithm used in this experiment allows to extract the importance of features based on a measure of “impurity.” That is, when training a single decision tree, we can compute how much each feature contributes to decreasing the weighted impurity, in our case measured with Gini impurity (Breiman, 2001). In the case of random forests, we measured the averaged decrease in impurity over trees to derive a feature importance score for each feature. To increase reliability we averaged these scores for each feature across the ensemble of 50 random forest classifiers in order to obtain the final scores for each word.

The scores for the nouns analysed in section 4.1 are presented in Table 1. The hypothesis is that nouns exhibiting the most temporal dissimilarities between the AD and non-AD classes identified in section 4.1 will also be used by the classifier to distinguish between the classes, resulting in larger feature importance scores. In this case, the sum of all three scores for each noun would give indication that the specific word appears in different sections of the transcript depending on the class to which the transcript belongs.

**Table 1:** Feature importance of nouns in a random forest classifier according to its position in 1st, 2nd, or 3rd chunk of each transcript.

**Noun**	**1st chunk**	**2nd chunk**	**3rd chunk**	**Sum**
Window	0.09904	0.02905	0.01041	0.13849
Sink	0.06526	0.03472	0.01101	0.11099
Stool	0.06090	0.02709	0.01988	0.10787
Action	0.07408	0.00796	0.00591	0.08795
Curtain	0.03686	0.02560	0.01131	0.07377
Mother	0.02548	0.01984	0.01852	0.06384
Dish	0.02689	0.01874	0.00951	0.05514
Cookie	0.03305	0.01190	0.00929	0.05424
Water	0.03082	0.01380	0.00704	0.05167
Hand	0.02241	0.01573	0.00780	0.04594
Girl	0.01303	0.01129	0.00828	0.03260
Boy	0.01023	0.00914	0.00903	0.02840
Jar	0.01080	0.00957	0.00724	0.02762
Plate	0.01398	0.00489	0.00475	0.02362
Floor	0.00970	0.00700	0.00651	0.02322
Kid	0.00787	0.00773	0.00566	0.02126
Thing	0.00657	0.00624	0.00424	0.01705
Sister	0.00870	0.00484	0.00112	0.01465
Lady	0.00482	0.00364	0.00264	0.01110
Kitchen	0.00578	0.00263	0.00217	0.01057

*Sum is the sum of all three scores*.

By measuring the Pearson correlation between the sums of scores (see column labelled “Sum” in Table 1) and differences in average position (column labelled “Difference” in Figure 1), we however obtain a weak non-significant negative correlation of –0.15 with a *p*-value of 0.53, indicating a possibility that the classifier considers more fine-grained temporal information, which is not visible by just averaging words' positions in the text. For example, the noun *window*, which was identified by the classifier as the most important feature out of all nouns in the list, does show a considerate difference in average position between AD and non-AD classes, but nevertheless still appears somewhere in the middle of the list in Figure 1. The same is true of the noun *sink*, which was identified as the second most important feature. Slightly more consistency between rankings can be observed at the bottom of both lists, for example when observing the ranking for nouns *kitchen* and *sister*.

## 5. AD Detection

The results of the temporal analysis in section 4 suggest that temporal differences in the descriptions can be detected in the transcripts and can also be successfully leveraged for detection of dementia by ML. Although it is doubtful that a classifier relying solely on temporal features would be able to achieve good performance, these features might improve AD detection when combined with other features. For this reason, in this section we explore a less specialised approach toward AD detection, which attempts to incorporate as many modalities and aspects of these modalities as possible. First, instead of focusing only on the textual information, we also incorporate several features extracted from audio modality, which are naturally time-based. As with audio, many aspects of the text modality are incorporated, including temporal, structural and semantic aspects.

### 5.1. Methodology

The main methodological steps of the proposed approach are described below, namely preprocessing, feature engineering and classification.

#### 5.1.1. Preprocessing

For audio preprocessing, speech segmentation was performed on the audio files that met the above described selection criteria. The study only focuses on the participants' speech; therefore, the investigators' speech was excluded from further processing. We extracted the participants' speech utterances using the timestamps obtained through DementiaBank.

The manual transcripts in CHAT format (MacWhinney, 2019) were first converted into word and token sequences which represent what was actually produced in speech. For instance, the annotations ‘w [x n]’, which indicate that the word ‘w’ was repeated n times were replaced by n repetitions of w, punctuation marks and various comments annotated between‘[]’ were removed. Also removed were symbols such as (.), (.), (…), <, <, / and xxx, as well as all punctuation.

Next, the processed transcripts were force-aligned with the speech recordings using the Penn Phonetics Lab Forced Aligner (Yuan and Liberman, 2008), which labels the pauses between words with ‘sp’ and produces time stamps for each word and for each pause. The word time stamps allowed us to split audio recordings at the level of words/pauses and conduct acoustic feature extraction for each word. The volume of each word was normalised to the range [−1: +1] dBFS. Volume normalisation helps in smoothing over different recording conditions, particularly variations in microphone placement in relation to the participant.

#### 5.1.2. Feature Engineering

**Figure 2:**
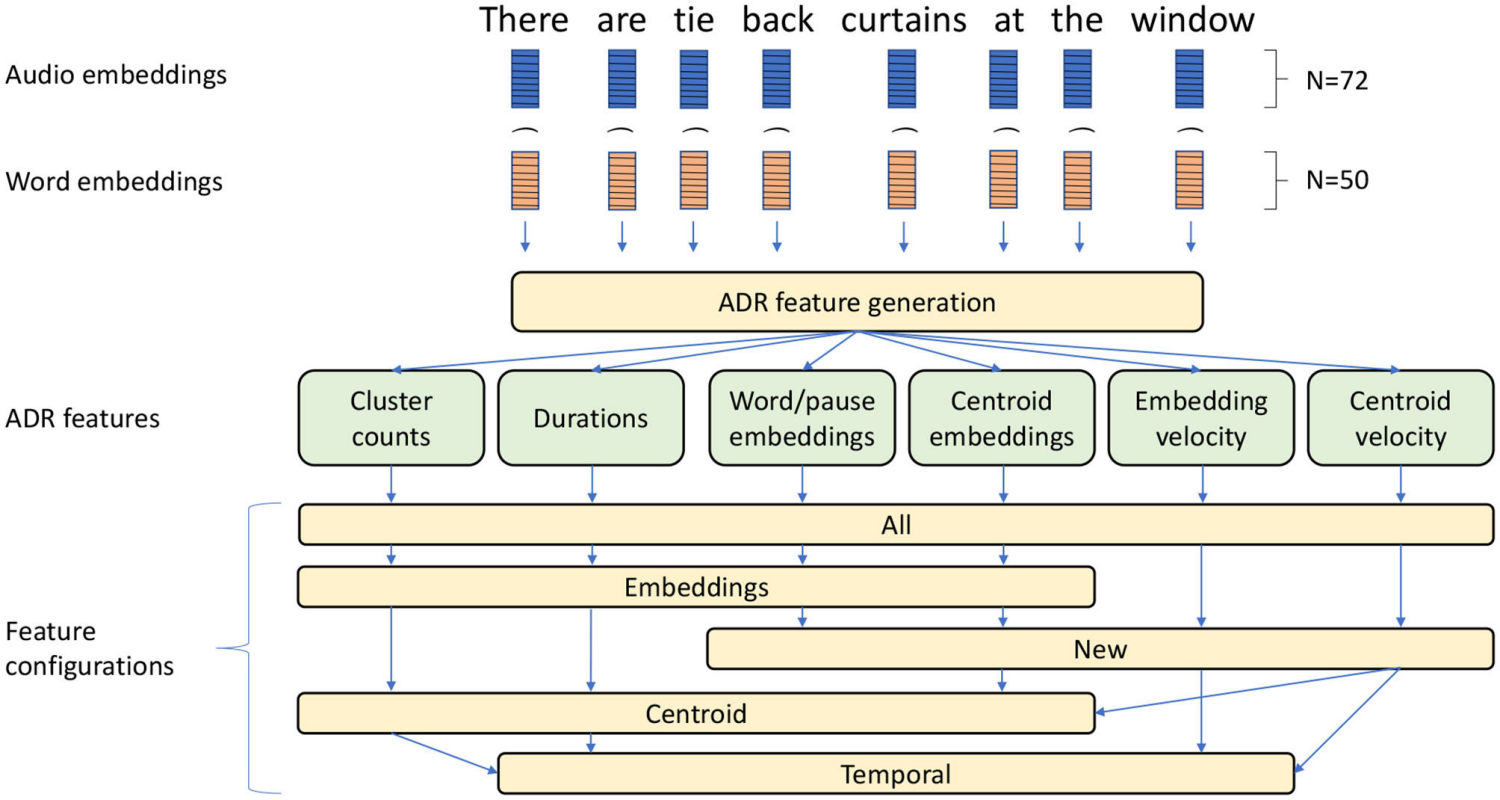
Main feature engineering steps presented on the example preprocessed input sentence “There are tie back curtains at the window”. Audio and word feature vectors (i.e., embeddings) are combined (Symbol “⌢” symbol denotes concatenations) and fed into an ADR feature generation procedure. The six resulting features are used in five distinct feature configurations.

The main steps of the feature engineering procedure are presented in Figure 2 and described below. The entire procedure can be divided into four main phases, generation and concatenation of audio and textual feature vectors, generation of six ADR features and selection of five distinct feature configurations.

The audio feature extraction was performed using the openSMILE v2.1 toolkit, which is an open-source software suite for automatic extraction of features from speech, widely used for emotion and affect recognition in speech (Eyben et al., 2010). In this research we opted to employ only the *eGeMAPS* (Eyben et al., 2016) feature set, which exhibited good performance in previous research (Haider et al., 2020). The *eGeMAPS* feature set corresponds to a basic set of acoustic features based on their potential to detect physiological changes in voice production, as well as theoretical significance and proven usefulness in previous studies (Eyben et al., 2016). It contains the F0 semitone, loudness, spectral flux, MFCC, jitter, shimmer, F1, F2, F3, alpha ratio, Hammarberg index and slope V0 features, as well as their most common statistical functionals, for a total of 88 features per speech segment. Pearson's correlation test was performed to remove acoustic features that were significantly correlated with duration (|*R*| > 0.2) to remove any bias toward the duration of words for machine learning. A total of 72 eGeMAPS features were therefore selected.

Following voice feature extraction we generated text features corresponding to the same words using GloVe embeddings (Pennington et al., 2014) of size 50 (for pauses, we generate a vector of 50 zeros). The audio and text features were normalised separately to the [0, 1] interval and concatenated to derive vectors of 122 features (72 audio features and 50 text features) corresponding to an audio-textual embedding for each word or pause. These vectors were then used in the ADR procedure for aggregation of words/pauses on the speaker level (Haider et al., 2020).

Note that in our implementation of ADR, we only loosely followed the original ADR algorithm, introducing several modifications. The procedure consists of the following steps:

1. **Clustering of feature vectors**: All word level feature vectors were aggregated into clusters using k-means clustering^4^. This is in contrast with the original implementation (Haider et al., 2020), which employed self-organising maps (SOM) clustering (Kohonen, 1990) but in line with the work done by Martinc and Pollak (2020).
2. **Generation of the ADR features**: The ADR feature vector is composed of several features, namely **cluster counts, duration, audio-textual word/pause embeddings, audio-textual centroid embeddings, audio-textual embedding velocity and audio-textual centroid velocity**. Note that the last four features were not employed in the original ADR (Haider et al., 2020) and are meant to also model the semantic aspects of the text input besides the temporal and structural properties of text and audio. Since the original ADR only modelled audio recordings, these features have not been used before. The following is a brief description of each of these features:
  - **Cluster counts**: Number of feature vectors in each cluster for each participant's audio recording, that is, a histogram of the number of words/pauses present in each cluster.
  - **Duration**: A histogram representation of word/pause utterance duration for each participant's audio recording. As the number and duration of segments varies for each audio recording, we normalised the feature vector by dividing it by the total duration of segments present in each audio recording.
  - **Audio-textual word/pause embeddings**: The audio-textual embeddings obtained for each participant were aggregated into a sequence. Principal component analysis (PCA)^5^ was conducted on the embedding sequence in order to reduce the dimensionality of each embedding to 1. Finally the sequence is truncated to the length of 128 if the sequence is too long, or padded with zeros if the sequence is too short. At the end of this procedure, we obtained a vector of 128 features for each participant.
  - **Audio-textual centroid embeddings**: Instead of employing PCA dimensionality reduction on audio-textual embeddings for each word, here we employed the procedure on the centroids of the clusters to which two consecutive word/pause utterances belong. At the end of this procedure, we obtained a vector of *k* features for each participant, where *k* is the number of clusters.
  - **Audio-textual embedding velocity**: In order to model temporal aspects of speech and transcripts, we measured the change between consecutive audio-textual embeddings in the sequence. This is measured with cosine similarity between consecutive vectors t and e:
    (2) $$\cos(t,e)=\frac{te}{\left\|t\|\|e\right\|}=\frac{\sum_{i=1}^{n}t_{i}e_{i}}{\sqrt{\sum_{i=1}^{n}{\left(t_{i}\right)}^{2}}\;\sqrt{\sum_{i=1}^{n}{\left(e_{i}\right)}^{2}}}$$
    The output of this procedure is a sequence of cosine distances between consecutive embeddings for each participant. The sequence was truncated (or padded with zeros) in order to obtain a vector of 128 features for each participant.
  - **Audio-textual centroid velocity**: Similarly, change is measured with cosine similarity between cluster centroids to which two consecutive word/pause utterances belong. The resulting sequence of cosine similarities was again truncated (or padded with zeros) to the length of 128.

To establish the contribution of specific features and to gain a better sense of what type of information results in the best performance, we tested several feature configurations:

- **Temporal**: Includes only four ADR features that model only temporo-structural aspects of the audio and transcript data, namely cluster counts, duration, audio-textual embedding velocity and audio-textual centroid velocity.
- **Embedding**: Includes four ADR features that model structural and semantic aspects of the data, namely cluster counts, duration, audio-textual word/pause embeddings and audio-textual centroid embeddings.
- **Centroid**: Includes four ADR features that model structural, semantic and temporal aspects of the data, namely cluster counts, duration, audio-textual centroid embeddings and audio-textual centroid velocity.
- **New**: Includes only the four new ADR features which have not been used in the previous studies where ADR was employed (Haider et al., 2020; Martinc and Pollak, 2020), namely audio-textual centroid embeddings, audio-textual centroid velocity, audio-textual word/pause embeddings and audio-textual centroid embeddings.
- **All**: Includes all 6 ADR features described in section 5.1.2.

In addition, we investigated the impact of specific input modalities on the overall performance, or to be more specific, we employed three versions for each of the configurations above:

- **Audio**: Only audio input is used, consisting of a feature vector for each word/pause containing only 72 eGeMAPS features.
- **Text**: Only text input is used, that is, a feature vector for each word/pause containing only 50 GloVe embeddings features. Here, there are also no Duration features, which require audio recordings for its generation.
- **Text+audio**: Combination of text and audio features, consisting of a feature vector for each word/pause containing 122 eGeMAPS and GloVe embeddings features.

Finally, we investigated if performance could be improved by adding sub-word units consisting of four-character sequences (char4grams) into the model. Even with the additional ADR features for modelling semantic aspects of the text, the initial experiments still suggested that semantic modelling might be the biggest shortcoming of ADR. It is indeed possible that the compressed semantic information obtained from word embeddings by employing clustering, PCA or cosine similarity is not comprehensive enough, since it models semantics (or semantic change) only indirectly. To compensate for this and model semantics more directly, in some experiments we employ term frequency-inverse document frequency (TF-IDF) weighted word bound character char4gram features, which proved very successful at modelling semantics in the study by Martinc and Pollak (2020). Character n-grams are created only from text inside word boundaries and n-grams at the edges of words are padded with space^6^.

#### 5.1.3. Classification

To determine the best classifier for the task at hand and the best number of clusters (*k*), we first conducted a preliminary grid search across several classifiers and *k* ∈ 10, 20, …, 80 values, in which we employed 5 classifiers from the Scikit library (Pedregosa et al., 2011), namely Xgboost (Chen and Guestrin, 2016) (with 50 gradient boosted trees with max depth of 10), random forest (with 50 trees of max depth of 5), SVM (with linear kernel and a box constraint configurations of 10), logistic regression (LogR, with a regularisation configuration of 10) and a linear discriminant analysis classifier. Only the *All* feature configuration was used during this preliminary experiment. Grid search was conducted on the training set, using LOOCV. Each classifier and *k*-value combination was run in the grid search five times, with five different random seeds for each classifier, in order to obtain more reliable results and to compensate for the observed variance in accuracy across different runs. The average accuracy across these five runs was used as a performance score for each combination of the classifier and *k*-value. Based on this score, the combination of k-means clustering with *k* = 30 and a random forest classifier was chosen for use in further experiments.

The large variance in accuracy (Equation 1) observed in these preliminary experiments is consistent with the observations of Yuan et al. (2020), where large variance in performance in the cross-validation setting was observed when employing BERT and ERNIE (Zhang et al., 2019) models. To solve this problem, they proposed a majority voting setting, in which the label assigned to an instance of the test set is the label assigned by the majority of the 50 models trained during cross-validation. We followed the same procedure and trained 50 models employing the same classifier and feature configuration on the training set, each time using a different random seed. These models were then used for majority voting on the test set to derive final predictions. The same procedure was employed to obtain comparable performance scores on the training set in LOOCV.

#### 5.1.4. Baseline BERT Implementation

In order to conduct the temporal experiments reported in section 6.1 and obtain a strong baseline, we also leverage the BERT model (Devlin et al., 2019). The preprocessing employed here was as described above, treating pauses as a form of punctuation, following Yuan et al. (2020). The transcripts were then force-aligned with the speech signal, labelling pauses between words with “sp', excluding pauses under 50 ms, and encoding short pauses (0.05–0.5 s) as ‘.’, medium pauses (0.5–2 s) as ‘.’, and long pauses (over 2 s) as '…'.

In contrast to Yuan et al. (2020), we fed the processed transcripts to the pretrained 'bert-base-uncased' language model with an additional linear sequence classification layer rather than the 'bert-large-uncased' model. This was done so as to reduce the amount of computational resources required. We did not employ the ERNIE (Zhang et al., 2019) language model, since the publicly available implementation of the model^7^ does not return the attention matrices required for the temporal analysis (see section 6.1). For fine-tuning, we employ the same hyperparameters as in the study by Yuan et al. (2020): learning rate = 2e-5, batch size = 4, epochs = 8, and maximum input length of 256. We set the standard BERT tokeniser not to split '…'.

Finally, we once again employed majority voting both in the LOOCV setting and on the test set. Due to limited computational resources, we only conducted the LOOCV procedure five times, with five different seeds, therefore obtaining five predictions for each example in the training set. The majority vote of these five predictions is used as a final prediction. On the other hand, for the test set setting, we randomly choose 50 models out of 540 models generated during LOOCV and conduct majority voting on the predictions of these models to obtain the final predictions.

## 6. Results

The results for the best feature combinations and input modalities are presented in Table 2. See Supplementary Material for a full table of results for all feature combinations (Supplementary Table 2), and for confusion matrices of the top 3 results and their late fusion (Supplementary Figure 2). For all results, we use k-means clustering with *k* = 30 and the random forest classifier, which yielded the best results in the preliminary grid search (see section 5.1.3).

**Table 2:** Results of the three best feature configurations in the LOOCV setting and on the test set in terms of accuracy.

**Feature configurations**	**Input modality**	**LOOCV accuracy**	**Test set accuracy**
Temporal + char4grams	audio + text	0.8611	**0.9167**
New + char4grams	audio + text	**0.8889**	0.8750
char4grams	text	0.8611	0.8958
top three late fusion	/	**0.8796**	**0.9375**
BERT—reimplementation of Yuan et al. (2020)	/	0.8426	0.8333
ERNIE best related work (Yuan et al., 2020)	/	/	0.8958

*The feature configurations column indicates which feature configuration has been used and whether char4grams have been added, and column Input modality shows the modality on which ADR features have been generated. The best individual methods' results in LOOCV and on the test set, as well as the late fusion of all three methods, are shown in bold. The row labelled top three late fusion presents the results of employing late/decision fusion (i.e., the use of majority voting) over the three best approaches*.

Without late/decision fusion of the best three methods, the best result on the test set was achieved when *Temporal* features generated on audio and text input were combined with *char4grams* (accuracy of 91.67%), and the best result in the cross-validation was achieved when *New* features generated on text and audio input were combined with *char4grams* (accuracy of 88.89%). *Char4grams* features by themselves also work very well, achieving an accuracy of 89.58% on the test set and accuracy of 86.11% in LOOCV. This indicates that semantic features and pause information contribute the most in terms of performance. Nevertheless, the results also indicate that we can improve the overall performance by including the temporal and structural aspects of audio and text.

Our reimplementation of BERT is noncompetitive in relation to the best approaches, reaching accuracy of 83.33% on the test set, which is in line with the results obtained by Yuan et al. (2020) who report accuracy of 85.4%. They however employ a larger BERT model with 24 layers and 16 attention heads for each layer.

**Figure 3:**
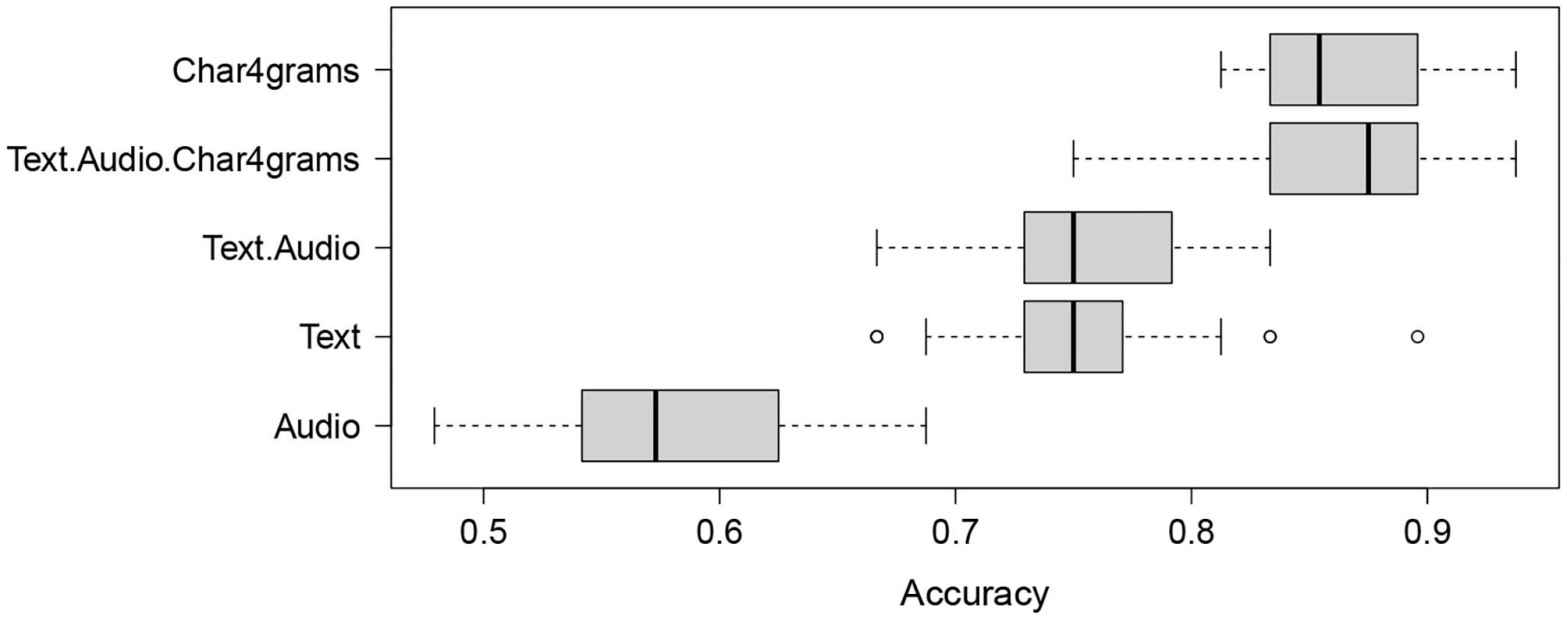
Boxplot summarizing the accuracy distributions for 50 classifiers on the test set for the *Temporal* feature configuration (text, audio, and text+audio), char4grams and char4grams combined with text and audio (text+audio+char4grams).

The observations from the error analysis (see Supplementary Material) suggest that employing late fusion can be beneficial. In our experiments it improved the best achieved test set accuracy of 91.67% by about 2.3% (to 93.75%) despite a slight decrease in accuracy in the LOOCV setting (from 88.89% to 87.96%). Another beneficial improvement is due to the use of majority voting, which reduces the variability of the test set predictions of single classifiers, shown in Figure 3. Figure 3 shows results of the accuracy distribution of 50 classifiers (employing temporal features and char4grams) used in the majority voting, when employed on the test set. It should be noted that the accuracy of 91.67% obtained by majority voting was obtained by <15% of classifiers in the ensemble, for the *temporal text+audio+char4grams* configuration. The other 85% of classifiers in the ensemble reach accuracy between 75 and 89%. Figure 3 also shows that the spread is largest when only the audio modality is used, ranging from about 48% to almost 70%.

The approach presented in this paper outperformed all previous approaches to AD detection performed on this and similar spontaneous speech datasets, as shown in Table 3. All accuracy figures for text correspond to accuracy on manual transcripts. Of the studies shown in Table 3, only Mirheidari et al. (2018) report results for embeddings derived from ASR transcription (62.5% accuracy), in contrast to the 75.6% they obtained from manual transcription. As noted, comparisons of studies done on different subsets and training/test splits of the Pitt corpus are problematic. The best previous result on the same dataset (ADReSS) used in our study was achieved by Yuan et al. (2020), who reported 89.58% test-set accuracy obtained with an ensemble of ERNIE models. Our late fusion method yielded an improvement of about 4.7% over the best reported result on the ADReSS dataset, and an improvement of 25% over the ADReSS challenge baseline (Luz et al., 2020).

**Table 3:** Comparison with state-of-the-art studies conducted on subsets of the Pitt dataset.

**Study**	**Accuracy**	**Modality**
Haider et al. (2020)	78.7%	Acoustic
Luz (2017)	68.0%	Acoustic
Fraser et al. (2016)	81.9%	Text/acoustic
Yancheva and Rudzicz (2016)	80.0%	Text/acoustic
Hernández-Domínguez et al. (2018)	68.0%	Text
Mirheidari et al. (2018)	75.6%	Text
**Studies based on the ADReSS dataset**		
ADReSS challenge baseline	62.5%	Acoustic
ADReSS challenge baseline	75.00%	Text
**Yuan et al. (****2020****) ERNIE**	**89.58%**	Text
Yuan et al. (2020) BERT	85.40%	Text
Syed et al. (2020)	85.42%	Text
Balagopalan et al. (2020)	83.33%	Text
Sarawgi et al. (2020)	83.33%	Text/acoustic
Pompili et al. (2020)	81.25%	Text/acoustic
Koo et al. (2020)	81.25%	Text/acoustic
Cummins et al. (2020)	81.25%	Text/acoustic
Searle et al. (2020)	81.25%	Text/acoustic
Edwards et al. (2020)	79.17%	Text/acoustic
Rohanian et al. (2020)	79.17%	Text/acoustic
Martinc and Pollak (2020)	77.08%	Text
Pappagari et al. (2020)	75.00%	Text/acoustic
***This study (best single model)***	***91.67%***	Acoustic/text/temporal
***This study (late fusion)***	***93.75%***	Acoustic/text/temporal

*The top three results are shown in bold. Results of this study are presented in Italics*.

### 6.1. Dissecting the BERT Attention Space

Another way to gain insight into how temporal information can be leveraged for AD detection, is through the use of neural networks, which model temporal and structural dependencies by default. The baseline BERT implementation described in section 5.1.4 is based on the transformer architecture, which employs the attention mechanism. The attention mechanism can be analysed and visualised, offering insights into the inner workings of the system. BERT's attention mechanism consists of 12 attention heads (Vaswani et al., 2017)—square matrices linking pairs of tokens within a given text. We explored how this (activated) weight space can be further inspected to establish to what extend BERT models temporal information.

While square attention matrices show the importance of the correlations between all tokens in the transcript, we focused only on the diagonals of the matrices, which indicate how much attention the model pays to a specific word in relation to itself, giving a measure of how important a specific word is for the classification of a specific description as belonging to either the AD or the non-AD class.

**Figure 4:**
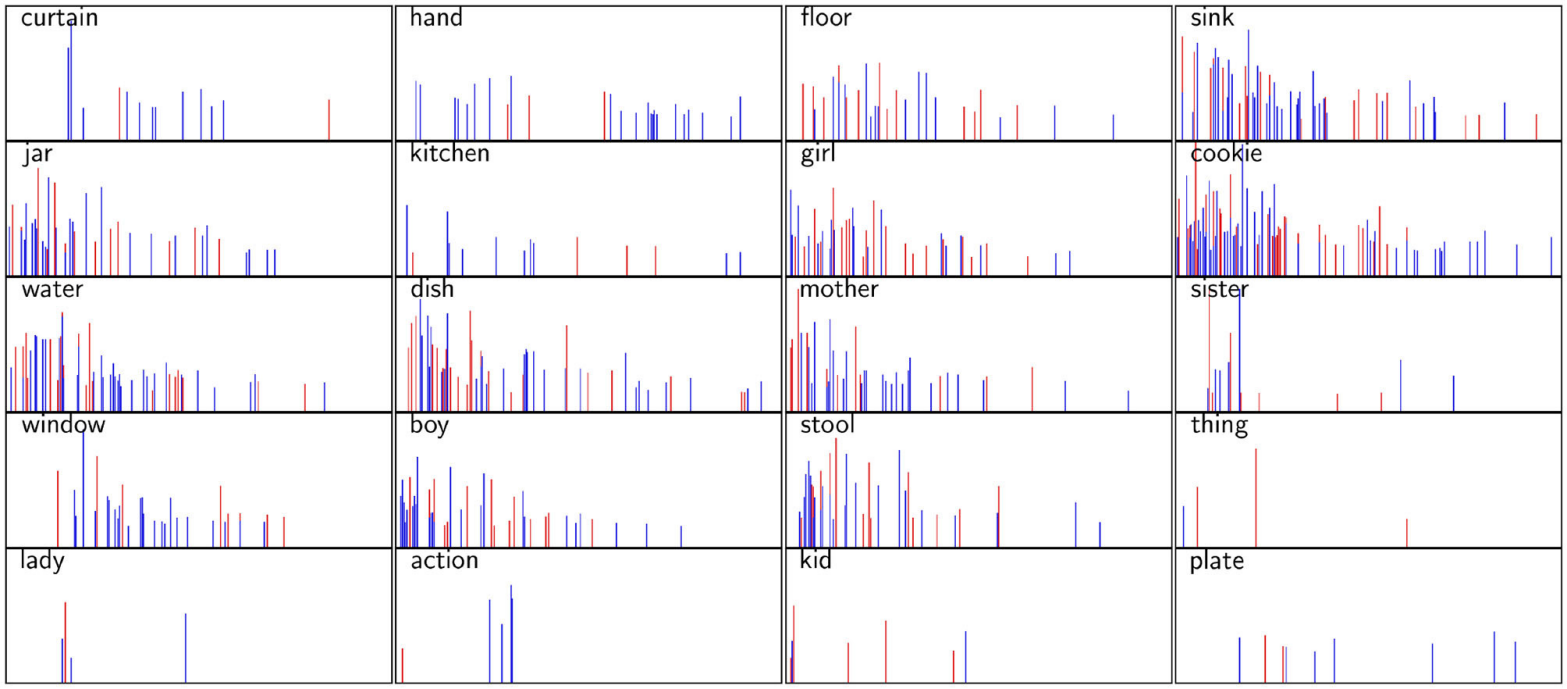
Test set attention scores prescribed by the BERT model for 16 nouns presented in Figure 1. The height of each column indicates the attention given to a specific noun in a specific position in the transcript. A blue coloured column indicates that a specific noun appeared in the transcript belonging to the non-AD class, while a red coloured column indicates that a noun at this position appeared in a transcript belonging to the AD class. The positions (*x*-axis) range from 1 (i.e., first word in the transcript) to 256 (i.e., last word in the corpus).

As explained in section 5.1.4, the BERT model was fine-tuned through LOOCV on the training set, and the fine-tuning procedure resulted in 50 BERT models, which were used for prediction on the test set. We extracted diagonal attention scores for 12 attention heads for each of the 20 nouns presented in Figure 1 appearing in different positions in different transcripts in the test set and averaged the scores across all 50 models. If a specific noun appeared in the same position in two or more different transcripts, scores belonging to the same position in each head were averaged. Finally, we also averaged the 12 attention heads scores for each position for each word so as to derive a sequence of attention scores for each noun. Figure 4 presents these sequences of attention scores for each of the 20 nouns appearing in different positions in the transcript. The height of each column indicates the attention given to a specific noun at position in the transcript, and the colour of the column labels the class of the transcript, blue denoting the non-AD class and red denoting the AD class.

Figure 4 shows that BERT generally tends to focus more attention to nouns appearing at the beginning of the transcript and less attention to nouns appearing at the end of the transcript. For example, for the noun *curtain*, attention scores are skewed toward the first few appearances of the word, dropping drastically afterwards. This suggests that the appearance of the word curtain in the last part of the transcript is not important for classification. A similar pattern can be discerned for the nouns *sister* and *window*. It can also be observed that some words (e.g., *hand, floor, kitchen* and *plate*) are not given as much attention as others, regardless of the position at which they appear.

While the attention scores derived from BERT suggest that the position of the word in the AD classification task does matter, there is no clear correlation between the attention scores given by BERT and the difference in average position for specific words identified in section 4.1. This might indicate that identification of temporal aspects is somewhat more involved than hypothesised, depending not only on the words' position but also on the context in which it appears.

## 7. Conclusions

We presented a study of automatic detection of AD in spontaneous speech using state-of-the-art ML methods. We conducted a temporal analysis of the descriptions of the Cookie Theft scene of the Boston Diagnostic Aphasia Exam (Goodglass et al., 2001) in order to investigate putative temporal differences between descriptions produced by AD and non-AD patients, and to explore the modelling of these differences by ML. We then proposed a new AD detection approach, in which ADR is employed as a framework for multimodal feature extraction and fusion. Through this approach our model was able to surpass the best state-of-the-art results reported in the literature for the task of distinguishing between transcripts and audio recordings belonging to AD and non-AD participants in the ADReSS subset of the Pitt Corpus.

While our models were able to distinguish between AD and healthy controls with relatively high accuracy using spontaneous speech data, further validation on larger and more diverse datasets is warranted. As pointed out by de la Fuente Garcia et al. (2020), datasets suitable for AI studies of the effects of neurodegeneration on spontaneously produced speech are relatively scarce at present. While this situation is changing, we hope our study will provide further impetus for research focused on elicitation and gathering of speech data from Alzheimer's cohort studies. An example of such studies is the PREVENT-ED spontaneous speech task, which has collected spontaneous dialogical speech from a group of healthy participants which includes participants genetically at-risk of AD, due to family history and apolipoprotein E (APOE) gene status (de la Fuente et al., 2019). Once the PREVENT-ED dataset has been fully collected, we aim to apply the methods presented in this article to investigate possible associations between speech features and the biomarkers available for the PREVENT cohort, including plasma and CSF Aβ42 amyloid, Tau and pTau, proinflammatory cytokines, acute-phase proteins, medial temporal-lobe atrophy and white matter lesion volume, as well as risk level (high, medium or low) and cognitive performance scores.

The results of the temporal bag-of-words model proved inconclusive in relation to the results of the analysis conducted in section 4.1. On the other hand, BERT results, while exhibiting sensitivity to temporal order, as words in different positions have different attention scores, are somewhat hard to interpret. These scores not only depend on temporal information but also indicate other differences between AD and non-AD patients related to semantic and grammatical contexts. We plan to address deficiencies of the temporal analysis and modelling in future work by investigating new temporal models and improving on our existing techniques for distillation of temporal information from the text.

Classification results indicate that accuracy gains can be achieved by adding temporal and structural information to semantic features. For example, the results show that the accuracy using only char4grams features (89.58%) can be improved to 91.67% when a combination of *temporal* audio textual features and char4grams features is employed, and up to 93.75% when late fusion of three best models is applied. These results compare favourably to the state-of-the-art. While these figures must be approached with caution given the relatively small size of the dataset, they provide motivation for further research into more challenging problems, such as earlier detection and prediction of AD progression, when suitable data become available in future.

Although the use of acoustic features on their own proved less successful than when combined with text, extraction of acoustic features can be fully automated, unlike textual features which if extracted through ASR would likely degrade classification accuracy. Therefore, while the multimodal approaches commonly employed in the recent ADReSS challenge (see Table 3) and extensively investigated in our study tend to benefit only marginally from the addition of acoustic information, processing and use of acoustic features is likely to remain an important topic of research in AD modelling, as will temporal aspects of spontaneous speech production.

As regards the use of transformer based embeddings, we believe they remain promising, despite their somewhat underwhelming contribution to classification performance in this study. Among other things, along with acoustic features, transformer based embeddings may play a role in the creation of language independent models for AD detection. Currently, multilingual BERT is being used in a variety of tasks allowing for classification to be performed on a language other than the language on which the model was trained (“zero-shot” transfer), and leveraging this possibility for cognitive decline detection would represent a valuable contribution to this field given that existing datasets are limited to only a few languages.

## Data Availability Statement

Publicly available datasets were analyzed in this study. The data can be found at https://dementia.talkbank.org. The source code for the experiments in this paper is available at https://github.com/matejMartinc/alzheimer_diagnosis.

## Author Contributions

SL, SP, FH, and MM conceived and designed the experiments and analysis. FH and SL compiled and prepared the dataset. MM and FH performed the analysis. SL and SP drafted the initial manuscript. All authors contributed to the final version of the manuscript.

## Conflict of Interest

The authors declare that the research was conducted in the absence of any commercial or financial relationships that could be construed as a potential conflict of interest.

## Footnotes

^1^The source code for the experiments and methods described in this paper is available under the terms of the MIT free software license at https://github.com/matejMartinc/alzheimer_diagnosis.

^2^The number of chunks was determined by finding the largest possible number of chunks where each set of chunks containing words from distinct positions in the text would contain at least one instance of each noun presented in Figure 1.

^3^Note that in this experiment we did not use term frequency-inverse document frequency (TF-IDF) weighting (Baeza-Yates and Ribeiro-Neto, 1999), as we did in the experiments in section 5.1 since we simply wanted the classifier to focus on binary differences between features (i.e., whether a specific temporal unigram appears in a transcript of a specific class or not).

^4^We use the Scikit library (Pedregosa et al., 2011) implementation of the algorithm: https://scikit-learn.org/stable/modules/generated/sklearn.cluster.KMeans.html

^5^Using the Scikit library implementation of the algorithm: https://scikit-learn.org/stable/modules/generated/sklearn.decomposition.PCA.html

^6^For example, for the sentence *It is sunny today*, the following set of char4grams would be generated: {“It,” “is,” “sun,” “sunn,” “unny,” “nny,” “tod,” “toda,” “oday,” and “day.”}.

^7^https://github.com/PaddlePaddle/ERNIE
