# Supplementary material for: Temporal Integration of Text Transcripts and Acoustic Features for Alzheimer's Diagnosis Based on Spontaneous Speech
Source: Front Aging Neurosci. 2021 Jun 14;13:642647. doi: 10.3389/fnagi.2021.642647 (PMC8236853; doi:10.3389/fnagi.2021.642647)
Supplement: Supplementary file 1 [file Data_Sheet_1.pdf]

Supplementary Material

1 SUPPLEMENTARY TABLES AND FIGURES

1.1 Explanation of selected terms

Active Data Representation (ADR): ADR is a feature extraction method for time-series data which generate a features vector of fixed dimension for large-scale datasets with variable duration Haider et al. (2020).
Area under the receiver operating curve (AUC): AUC is a performance measurement for classification, which provides an aggregate measure across all possible classification thresholds.
Attention: In deep neural networks, attention is a component of a network's architecture responsible for managing and quantifying the interdependence between input and (optionally) output elements. In the paper, the term attention in all cases refers to the so-called self-attention, which only quantifies dependencies between input elements (e.g., dependencies between words in the input text), in contrast to the general attention, which considers dependencies between input and output elements.
Bag-of-words (BoW), Bag-of-n-grams: BoW refers to the text representation method, where a document (an instance) is represented by a set of (possibly weighted) frequencies belonging to words in this document and the word order is disregarded. Bag-of-n-grams similarly considers frequencies, but instead of considering only words, also bigrams, trigrams or sequences of more words are considered. Similarly to words, other text levels can be considered, e.g. character n-grams.
BERT: BERT stands for Bidirectional Encoder Representations from Transformers (Devlin et al., 2019). It is a neural network based on the Transformer architecture (Vaswani et al., 2017) pretrained on a large corpus of text in an unsupervised way as a masked language model in order to learn contextual text representations. The model can be fine-tuned for a specific classification task.
Classifier: A machine-learning algorithm used to build a classification model from training data.
Clustering: Clustering is the task of grouping a set of objects into groups, in which the objects are more similar to each other than to those in other groups.
eGeMAPS: The *eGeMAPS* feature set (Eyben et al., 2016) resulted from an attempt to reduce the somewhat unwieldy audio feature sets to a basic set of acoustic features based on their potential to detect physiological changes in voice production, as well as theoretical significance and proven usefulness in previous studies. It contains the F0 semitone, loudness, spectral flux, MFCC, jitter, shimmer, F1, F2, F3, alpha ratio, Hammarberg index and slope V0 features, as well as their most common statistical functionals, totalling 88 features.
Ensemble: Ensembles refers to combining multiple algorithms for a machine learning task.
Features: Features are variables that are used for describing a training or test set instance for a machine learning algorithm. An example of a simple feature is frequency of a word in a document, but features can also be more complex, especially when resulting from sophisticated feature engineering techniques.
GloVe embeddings: GloVe embeddings are multi-dimensional vector representations of words (embeddings) that were trained using GloVe (from Global Vectors), an unsupervised learning algorithm. Training is performed on aggregated global word-word co-occurrence statistics from a corpus, and the resulting representations showcase interesting linear substructures of the word vector space. The global logbilinear regression model combines the advantages of global matrix factorization and local context window methods (Pennington et al., 2014).
Late fusion: While early fusion describes merging features of different models before classification, late

fusion indicates combining the outputs of different classification models.
Leave-one-out cross-validation (LOOCV): Cross-validation refers to machine-learning model evaluation approach, where training data and test data are rotating. In leave-one-out cross-validation setting, all the instances but one are taken as training data, and the left-out one serves as test data, and the process is repeated as many times as the number of instances.
Majority voting: It refers to ensemble methods in machine learning, where several models are making prediction for the test instance, and the final prediction for an instance is the one which achieves most of the votes by different models.
Random forest classifier: A random forest is an ensemble of decision tree classifiers, which are fitted on various sub-samples of the dataset. The predictions of these classifiers are averaged to improve the predictive accuracy and control the over-fitting of the final ensemble model.
Spectral subtraction is a background acoustic noise reduction method for audio signal.
Statistical functionals are functions of probability distribution functions. Some examples of statistical functionals are mean, standard deviation, minimum and maximum values over a time period.
Support vector machine (SVM): A Support Vector Machine (SVM) is a supervised learning classifier that given a labelled training data outputs an optimal hyperplane which serves for separating new examples into predefined classes.
Training set and test set: In machine learning, the data set is split into train set used to train the model, and the test set, which is not used during the training and serves for unbiased model evaluation. In some cases, for an intermediate evaluation, and additional validation set is used for tuning the model's hyperparameters.

2 DATA PROCESSING AND ADRESS DATASET DETAILS

Table S1. ADReSS dataset (Luz et al., 2020): Basic characteristics of the patients in each group (M=male and F=female).

Training set						
	AD			non-AD		
Age	M	F	MMSE (sd)	M	F	MMSE (sd)
$[50, 55)$	1	0	30.0 (n/a)	1	0	29.0 (n/a)
$[55, 60)$	5	4	16.3 (4.9)	5	4	29.0 (1.3)
$[60, 65)$	3	6	18.3 (6.1)	3	6	29.3 (1.3)
$[65, 70)$	6	10	16.9 (5.8)	6	10	29.1 (0.9)
$[70, 75)$	6	8	15.8 (4.5)	6	8	29.1 (0.8)
$[75, 80)$	3	2	17.2 (5.4)	3	2	28.8 (0.4)
Total	24	30	17.0 (5.5)	24	30	29.1 (1.0)
Test set						
	AD			non-AD		
Age	M	F	MMSE (sd)	M	F	MMSE (sd)
$[50, 55)$	1	0	23.0 (n.a)	1	0	28.0 (n.a)
$[55, 60)$	2	2	18.7 (1.0)	2	2	28.5 (1.2)
$[60, 65)$	1	3	14.7 (3.7)	1	3	28.7 (0.9)
$[65, 70)$	3	4	23.2 (4.0)	3	4	29.4 (0.7)
$[70, 75)$	3	3	17.3 (6.9)	3	3	28.0 (2.4)
$[75, 80)$	1	1	21.5 (6.3)	1	1	30.0 (0.0)
Total	11	13	19.5 (5.3)	11	13	28.8 (1.5)

3 RESULTS FOR ALL FEATURE CONFIGURATIONS

The results for all feature combinations and input modalities are presented in Table S2. We also present confusion matrices of top 3 results (feature configurations shown in boldface in Table S2) and their late/decision fusion in Figure S2. Different configurations of ADR features perform adequately on their own when generated on text and audio modalities, yet still much worse than in combination with *char4grams*. When used on their own, the best ADR feature configuration is *Embedding* employed only on the text

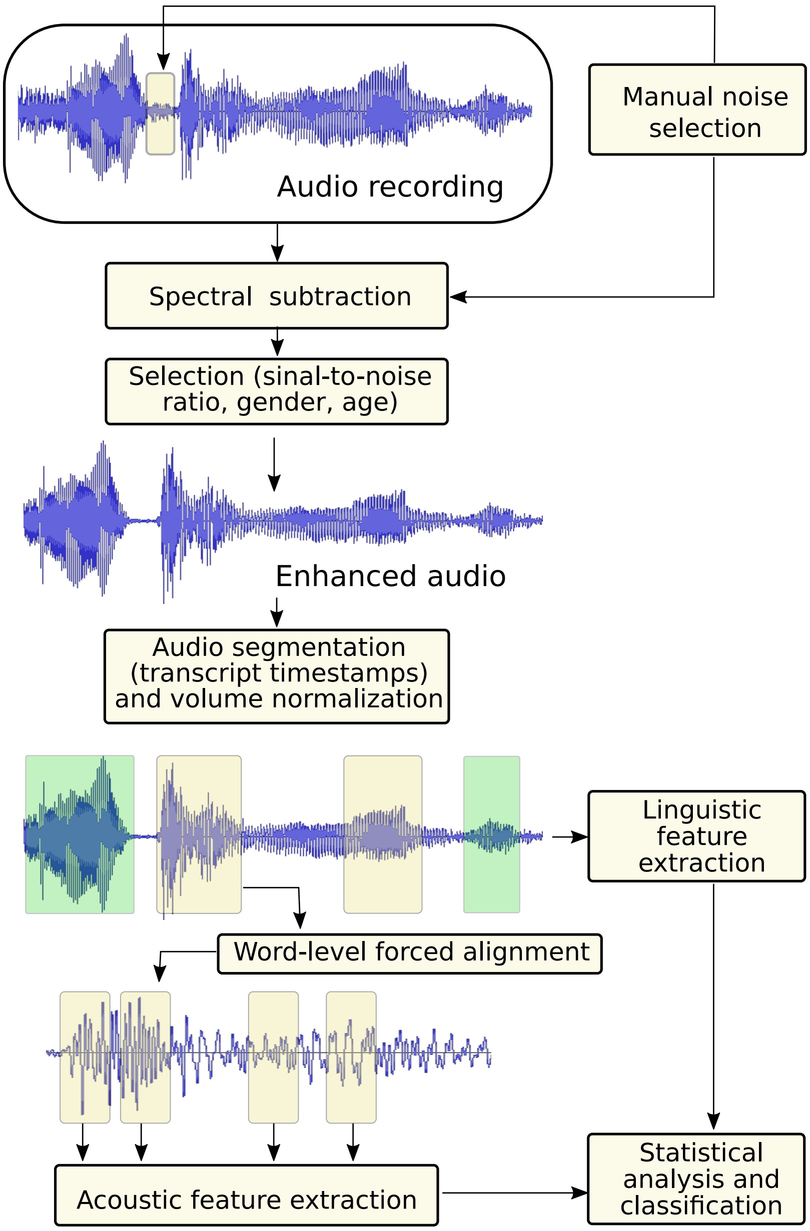

Figure S1. The ADReSS data processing pipeline. Acoustic preprocessing is shown on the top part of the diagram. Noise was sampled from short intervals from each audio recording and subsequently eliminated through spectral subtraction. Other non-target sounds such as background talk, ambulance sirens and door slamming, were minimised by selecting audio files with signal-to-noise ratio (SNR) greater than or equal to -17 dB.

modality, which achieves 87.50% accuracy on the test set. This is in line with the good performance of *char4grams*, since this configuration is the most focused on modelling semantics out of all ADR feature configurations.

When a combination of audio and text modalities is used as input, the best configurations in terms of performance are *Temporal* and *Centroid*, achieving the accuracy of 79.17% on the test set. The worst performance when used on their own is observed for the *New* feature configuration, where accuracy on the test set drops to 70.83%, when text and audio modalities are used, and to 72.92%, when only the

Table S2. Results in the LOOCV setting and on the test set in terms of accuracy. While column Feature configuration indicates which feature configuration has been used and whether char4grams have been added, column Input modality shows the modality on which ADR features have been generated. The top three methods' results in leave-one-out cross-validation (LOOCV) settings are in bold including their performance on test data. Line 'top three late fusion' presents the results of employing late/decision fusion (i.e., the employment of majority voting) over the three best approaches.

Feature configuration	Input modality	LOOCV accuracy	Test set accuracy
Temporal	audio	0.5185	0.6458
Temporal	text	0.6574	0.7917
Temporal	audio + text	0.6667	0.7917
Temporal + char4grams	audio + text	**0.8611**	**0.9167**
All	audio	0.5648	0.5625
All	text	0.7037	0.8542
All	audio + text	0.6852	0.7708
All + char4grams	audio + text	0.8241	0.8750
Embedding	audio	0.5833	0.6042
Embedding	text	0.7037	0.8750
Embedding	audio + text	0.6944	0.7708
Embedding + char4grams	audio + text	0.8333	0.8542
Centroid	audio	0.5926	0.5833
Centroid	text	0.6852	0.7917
Centroid	audio + text	0.6852	0.7917
Centroid + char4grams	audio + text	0.8519	0.8750
New	audio	0.5926	0.5000
New	text	0.6667	0.7292
New	audio + text	0.6204	0.7083
New + char4grams	audio + text	**0.8889**	**0.8750**
char4grams	text	**0.8611**	**0.8958**
top three late fusion	/	***0.8796***	***0.9375***
BERT - reimplementation of Yuan et al. (2020)	/	0.8426	0.8333
ERNIE best related work (Yuan et al., 2020)	/	/	0.8958

text modality is used as input. This indicates that structural information obtained from the cluster counts contributes disproportionately more to the overall classification accuracy than other features.

Audio features by themselves perform rather poorly, reaching accuracy of up to about 65% on the test set. When combined with textual features, they improve the performance of the classifier just when the *New* configuration is employed, by roughly 2 percentage points. On the other hand, in most cases, the performance of the classifier drops when audio features are used. The drop is the biggest for the *All* configuration, about 10 percentage points on the test set.

4 ERROR ANALYSIS

To observe the relationship between the top three methods, and the potential improvement that might be obtained by combining the outputs of different models (late fusion), we drew the Venn diagrams as shown in Figure S3 and Figure S4 for validation and test datasets respectively. In Figure S3 and Figure S4, the blue area (Target) represents the annotated labels, the yellow ellipse represents the predicted labels by the *temporal audio + text + char4grams* feature set, the green area represents the predicted labels by the *no count + duration audio + text + char4grams* feature set, the red area represents the predicted labels by the *char4grams* feature set, and finally the brown area represents the labels predicted by late fusion.

From the overlaps in Figure S3 (validation data), it is observed that there are 6 instances (4 of AD and 2 of non-AD) which have not been recognised by any of the feature sets. In contrast, there are 85 instances (45 of non AD and 40 of AD) which have been detected by all four methods. While a detailed analysis of misclassified examples is beyond the scope of this study, we noticed that from the four misclassified AD

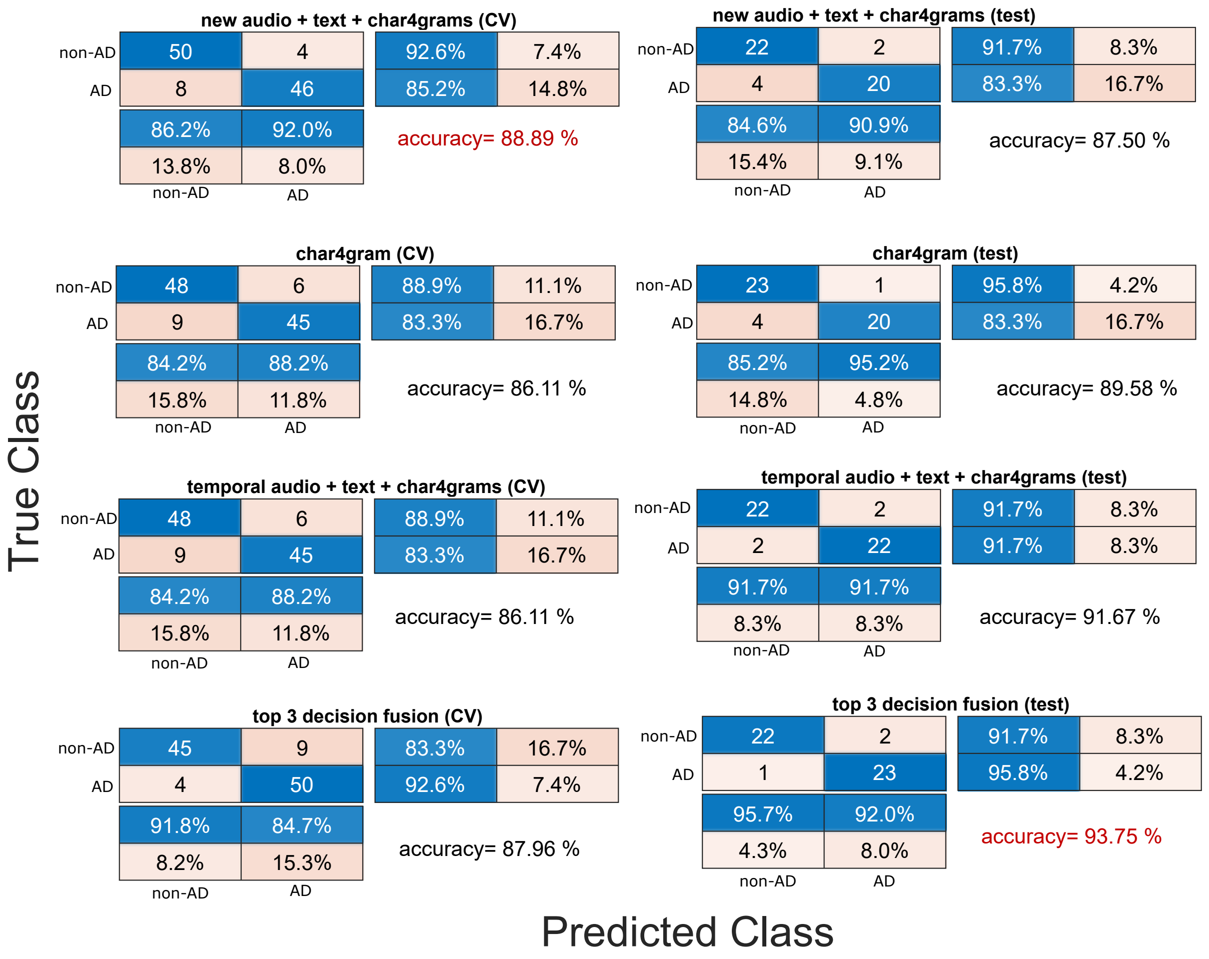

Figure S2. Confusion matrices for leave-one-out cross-validation (CV) and test set results of top three models and their late fusion (see confusion matrices titled *top 3 decision fusion*). Confusion matrices titled *new audio + text + char4grams (CV)* present results for the new ADR feature configuration employed on audio and text modalities with added char4grams features, confusion matrices titled *temporal audio + text + char4grams* presents results for the *temporal* ADR feature configuration employed on audio and text modalities with added char4grams features, and confusion matrices titled *char4gram* present results when only char4gram features are used.

instances, three have relatively high MMSE scores (23, 24 and 27) and one has a "possible AD" diagnosis (as opposed to "probable AD", which is the diagnosis assigned to all other patients in the AD class), which might indicate that these were indeed borderline cases. On the test data, only two instances (of AD and of non-AD) were missed by all four methods, against 40 instances correctly detected by all four methods. The *temporal audio + text + char4grams* (91.67%) configuration provides slightly better results than *new audio + text + char4grams* (87.50%) and *char4grams* (89.58%) but the last two configurations are nevertheless able to capture different information as shown in Figure S4.

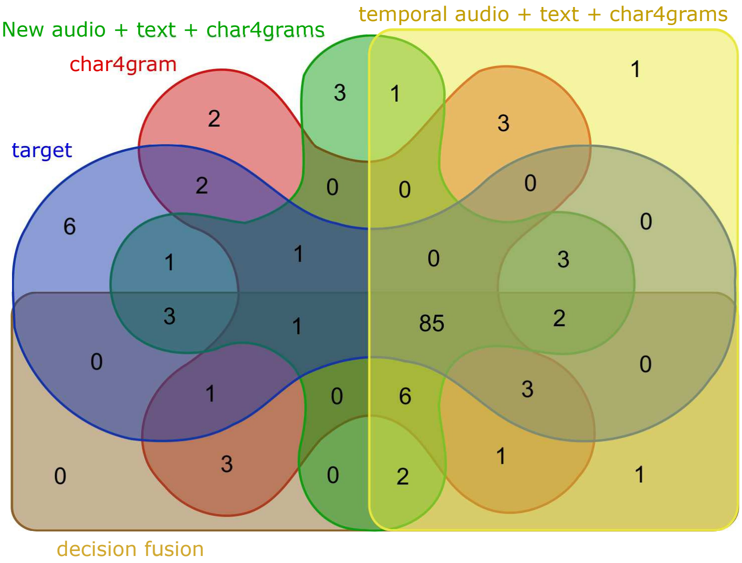

Figure S3. Venn Diagram of top three models and their late fusion in LOOCV. It represents mutual agreement between methods. Each coloured area represent a method, and the blue area represents the ground truth ('target'). The number within coloured areas represents the number of instances.

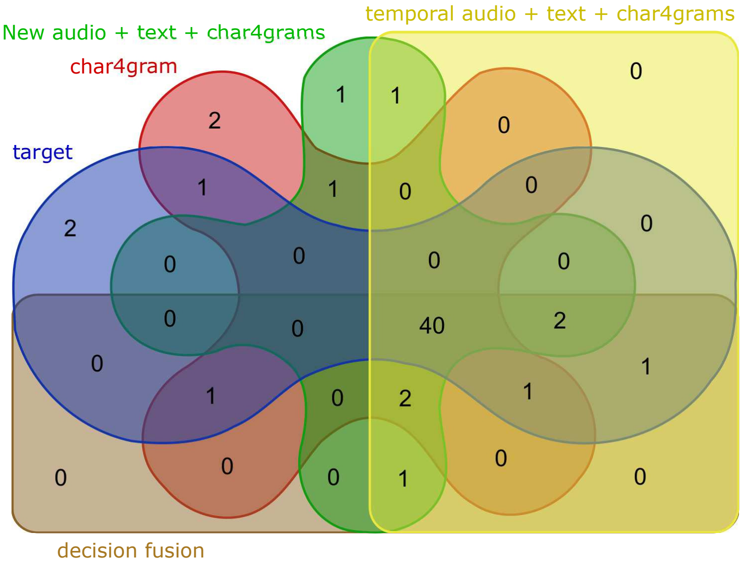

Figure S4. Venn Diagram of top three models and their late fusion on the test set. It represents mutual agreement between methods. Each coloured area represent a method, and the blue area represents the ground truth ('target'). The number within coloured areas represents the number of instances.

REFERENCES

Devlin, J., Chang, M.-W., Lee, K., and Toutanova, K. (2019). BERT: Pre-training of deep bidirectional transformers for language understanding. In *Proceedings of the 2019 Conference of the North American Chapter of the Association for Computational Linguistics: Human Language Technologies, Volume 1* (Minneapolis, Minnesota: Association for Computational Linguistics), 4171–4186. doi:10.18653/v1/N19-1423

Eyben, F., Scherer, K. R., Schuller, B. W., Sundberg, J., André, E., Busso, C., et al. (2016). The Geneva minimalistic acoustic parameter set GeMAPS for voice research and affective computing. *IEEE Trans. Affect. Comput."* 7, 190–202

Haider, F., de la Fuente, S., and Luz, S. (2020). An assessment of paralinguistic acoustic features for detection of Alzheimer's dementia in spontaneous speech. *IEEE Journal of Selected Topics in Signal Processing* 14, 272–281. doi:10.1109/JSTSP.2019.2955022

Luz, S., Haider, F., de la Fuente, S., Fromm, D., and MacWhinney, B. (2020). Alzheimer's dementia recognition through spontaneous speech: The ADReSS challenge. In *Proceedings of Interspeech 2020*. 2172–2176. doi:10.21437/Interspeech.2020-2571

Pennington, J., Socher, R., and Manning, C. D. (2014). GloVe: Global vectors for word representation. In *Proceedings of the 2014 conference on empirical methods in natural language processing (EMNLP)*. 1532–1543

Vaswani, A., Shazeer, N., Parmar, N., Uszkoreit, J., Jones, L., Gomez, A. N., et al. (2017). Attention is all you need. In *Advances in neural information processing systems*. 5998–6008

Yuan, J., Bian, Y., Cai, X., Huang, J., Ye, Z., and Church, K. (2020). Disfluencies and fine-tuning pre-trained language models for detection of Alzheimer's disease. In *Proceedings of Interspeech 2020* (Shanghai, China), 2162–2166
